# The Use of Soft Foil

**Published:** 1898-04

**Authors:** C. C. Chittenden

**Affiliations:** Madison, Wis.


ARTICLE VIII.

### THE USE OF SOFT FOIL.

BY C. C. CHITTENDEN, D. D. S., MADISON, WIS.

**Read at Twenty-Seventh Annual Meeting of Wisconsin State Dental Society, July 20-22, 1897.**

Up to less than forty years ago the only form in which gold was used for filling teeth by all the dentists of the world was soft foil. If the foil "stuck together" or acted "stiff," i. e., was not entirely free from what we now know to be "cohesive properties," it was at once condemned as unfit for use and "probably not properly refined." The gold beaters had great difficulty in overcoming this objectionable property and often had their work to do over again because of its presence. The only way fillings were made was on the wedge principle of impacting the soft and ductile foil from wall to wall of a cavity so shaped as to be of a general retaining form.

The plugging instruments used, of all shapes and forms, with whatever kind of points, were always perfectly smooth and polished throughout, with large handles capable of being firmly grasped by the full hand, so that great force and pressure could be brought to bear to "pack" the gold against the cavity walls. The commonest form of gold used was about No. 6 to 10 foil rolled into "rope" form. If the cavity was a molar crown, for instance, it would be shaped with nearly perpendicular walls, no overhangs, and the mouth of the cavity slightly larger than the floor.

The end of the rope was firmly folded over the end of a proper sized plugger and expertedly packed and fastened into a convenient angle or notch, the plugger withdrawn and caught into the rope far enough back to bring down the main rope into the cavity beside the end already secured, forming a loop long enough to overhang the depth of the cavity from one-sixteenth to one eighth of an inch. This was repeated until the circuit of the cavity was made, each fold being firmly packed against the preceding one. Then a plugger as large as admissible, with a flat, smooth end, was forced to the bottom in the center and pressure brought to bear in every direction toward the side walls, firmly forcing the loops standing on end against them. This had the effect of forcing the overhanging ends of the loops over the edges of the cavity. Next a large firm roll or pellet of foil was forced into the central pit, then a smaller wedge point was forced in beside it, and so long as an opening could be made other and smaller pellets were forced into the general wedge.

When this wedging process was complete, the next step was to condense down the overhanging ends of the loops. This was a very important part of the operation and demanded much skill. First a flat-faced smooth plugger (and all pluggers were smooth) condensed the gold straight on end. This left the marginal gold overlying the wall edges. This marginal surplus was then cunningly coaxed toward the center until, if the proper proportion of overhanging gold had been maintained, a perfect and solid margin resulted. In all this process nothing had been done to mar or tear or in any way disturb the integrity of the heavy soft foil, and there was a solid, impervious filling as a result, without the slightest danger of any flaking or disintegration.

Of course it was thought best if possible to keep things dry during all this time, but if moisture or a flood did break in the operator went right on, after letting the patient "spit out."

It mattered little where the cavity was located. The aim in preparation was to secure walls as firm and strong as possible, which continued of nearly equal proportions throughout the whole circumference of the cavity, so that the wedge fastening was complete. To accomplish this the file was a constant necessity. If the proximate surface of a bicuspid or molar was involved and the cavity encroached on the grinding surface, the V file was freely used until even the wall toward the crown surface was firm enough to be wedged against. This left of course a large space between the teeth, just the opposite of what we are to-day taught to consider correct, i. e., the nearest point of contact with the adjoining tooth was next the cervical border and not near the now-called occlusal surface.

It was noticeable too that the file left its trail of lines along the sides of the V, and there was no engine with sandpaper disks to polish them out, but for some unaccountable reason a large percentage of these soft-foil wedge fillings have stood their ground, and have been seen in many a mouth of the passing generation like the Sphynx calmly surveying a surrounding desert waste.

Another form of using soft gold in these cavities was to fold the foil into ribbons of a width one-third greater than the depth of the cavity and roll this ribbon on a square drill point into cylinders of different sizes and density. These were introduced on end and packed against each other around the walls like the loops spoken of, and the final wedges inserted in the center, then condensing the whole on end.

Now, gentlemen, I have told of all this not to try to convince anybody that it is the best and only way to fill and save teeth, but to call attention to a property in gold that I feel is not given enough attention to-day in college training, where young men are supposed to be fully prepared and equipped to do all things required of them in the best and wisest way. Soft gold is susceptible of being brought into perfect contact and adaptation to the side and floor walls of proximate cavities of molars and bicuspids by the wedging process and the burnisher—*more* perfect contact with far less danger of injury to delicate and frail margins of such cavities than the harder and less plastic cohesive forms of gold in general use. With the latter the mallet is almost indispensable, and in many cases the result of its use is to check, mar and even pulverize the enamel border, thus, although not visible to the naked eye, insuring certain ultimate failure of the operation. This class of cavities is in my judgment least likely to failure by the use of soft gold for the proximate portion, finished toward the grinding surface with any form of cohesive gold desired.

The two best ways of making such fillings, are. First, Use a band matrix not too closely fitting the walls, and so force and wedge soft foil against the walls and matrix as to cause a surplus or overhanging of gold throughout the proximate borders which, after removal of matrix, must be burnished carefully over the margins and properly finished and polished. Second: Make cylinders of soft foil as before described, one-fourth to one-third longer than the lateral depth of the cavity, and so place and wedge them against the floor and walls that their overhanging ends extend into the space, there to be later burnished against and over the enamel margins. It is the simplest matter in the world to complete these operations with cohesive gold, beginning at any point from which forward it is desirable to have a hard welded surface for mastication.

Do not understand me to claim that this is the only successful method of adapting gold to proximal margins be thoroughly and intelligently pursued, a permanent success is more likely to result than where indiscriminate and unmerciful mallet-pounding is used to adapt stiff and hard cohesive gold to frail enamel margins.

### DISCUSSION.

Dr. R. G. Richter: I always use soft foil in proximal cavities, because the edges can be burnished over so nicely. I have seen fillings that have stood the test of time, while fillings in the same mouth made with cohesive foil failed simply because of the breaking down of the enamel rods from the force used in condensing.

Dr. R. Maercklein: Cohesive foil may be just as soft as non cohesive. The difference is, that the latter will not cohere, while the former will stick just where it is placed. If you could key non-cohesive foil there would be no necessity of finishing off the filling with cohesive. If you put enough pressure on it about the same as tin foil—simply wedge it in.

Dr. B. G. Maercklein: There is a good reason why the work done by the older practitioners stood the test of time better than that performed by the dentists of the present day. The former class were skilled. They had trained their hands and eyes to a degree of perfection seldom reached by the present generation of dentists. And Why? Because they had to. With the newer appliances and improvements, such as rubber dam, dental engines, mallets and cohesive gold, have come into the profession a line of inferior skill and deficient development in manipulative ability. The cause of one-half the failures to-day is not the material but the manner in which it is used.—*Dental Digest.*

